# Multiple functions of hypoxia-regulated miR-210 in cancer

**DOI:** 10.1186/1756-9966-33-50

**Published:** 2014-06-09

**Authors:** Qin Qin, Wei Furong, Li Baosheng

**Affiliations:** 1 Department of Radiation Oncology, Shandong Cancer Hospital, Shandong University, Jiyan Road 440, Jinan 250117, P.R. China; 2 Department of Radiation Oncology, Shandong Cancer Hospital, Shandong Academy of Medical Sciences, Jinan 250117, P.R. China; 3 Institute of Basic Medicine, Shandong Academy of Medical Sciences, Jingshi Road 18877, Jinan 250062, P.R. China

**Keywords:** microRNA, Hypoxia, miR-210, Proliferation, Apoptosis, Angiogenesis, Metabolism, Diagnosis, Prognosis

## Abstract

MicroRNAs (miRNAs) are small noncoding RNAs that regulate gene expression post-transcriptionally. miRNAs can be induced by a variety of stresses such as hypoxia, and are involved in diverse biological processes including differentiation, cell proliferation, cell death, and tumorigenesis. Hypoxia, a common feature of tumor microenvironment, can induce a number of miRNAs expression. miRNA-210 (miR-210) is one of the hypoxia-regulated-miRNAs, which has been investigated extensively in cancer. However, paradoxically opposing results were documented regarding whether it is an oncogene or a tumor suppressor, and whether it is a positive or negative prognostic biomarker. In the present review, we focus on the following investigations of miR-210: 1) its functions of as an oncogene, 2) its functions as a tumor suppressor, 3) its functions in mitochondrial metabolism, and finally, the diagnostic and prognostic value of miR-210 in cancer researches.

## Introduction

MicroRNAs (miRNAs) are small noncoding RNAs that regulate gene expression post-transcriptionally by pairing to 3’ untranslated regions (UTRs), coding sequences or 5’ UTRs of target messenger RNAs (mRNAs), which in most cases leads to translation inhibition or mRNA degradation [1]. In mammals, miRNAs are predicted to regulate the activity of approximately 50% of all protein-coding genes [2]. Due to the widespread regulating functions, miRNAs are involved in almost every cellular process including differentiation, cell proliferation, cell death, and tumorigenesis [3].

Hypoxia is a common feature of the tumor microenvironment [4] and has been an extensively investigated field in cancer researches demonstrating its critical role in various physiologic and pathologic processes including cell proliferation, cell survival, angiogenesis, metabolism, tumor invasion and metastasis [5]. It is widely accepted that hypoxia represents an independent adverse prognostic factor in many tumor types [4,6].

Since the first article demonstrated the functional link between hypoxia and miRNAs expression, which identified a specific hypoxia-regulated miRNAs (HRMs) playing an important role in cell survival in low oxygen environment [7], that more and more HRMs were identified [8-12]. Although discrepancies exist among HRMs identified by different research groups, the up-regulation of miR-210 induced by hypoxia has been consistent in all published studies in both normal and transformed cells, which implies an essential role of miR-210 for cell adaptation to hypoxia [13-15]. Not only in vitro studies correlated miR-210 with hypoxia, in vivo investigation also verified it. In tumor tissues such as breast cancer and head and neck cancers, miR-210 expression levels have been demonstrated to be correlated with hypoxia gene signatures, which suggested a direct connection between miR-210 expression and hypoxia [16,17].

miR-210 is an intronic miRNA located within the genomic loci of transcript AK123483 [18]. While most studies reported miR-210 regulation in a hypoxia-inducible factor-1 (HIF-1)-dependent way [19-21], HIF-2-dependent [22,23] and HIF-independent [24,25] regulation of miR-210 have also been reported. The master HRM miR-210 has been investigated intensively, which has identified a variety of functionally important targets involved in cell cycle regulation [18,22,26-30], cell survival [31-36], differentiation [37-40], angiogenesis [41-51] as well as metabolism [52-57].

However, paradoxically opposing results were documented with regard to whether miR-210 is an oncogene or a tumor suppressor, and whether it is a positive or negative prognostic biomarker. In the present review, we focus on the following investigations of miR-210: 1) its functions of as an oncogene, 2) its functions as a tumor suppressor, 3) its functions in mitochondrial metabolism, and finally, the diagnostic and prognostic value of miR-210 in cancer researches.

### miR-210 functions as an oncogene

Since miR-210 is up-regulated ubiquitously and robustly in hypoxic cells and hypoxia is a key feature of solid tumors, it is reasonable to explore the functions of miR-210 in tumorigenesis. With the development of bioinformatic miRNA targets prediction tools as well as the improvement of experimental approaches, many diverse targets of miR-210 have been identified, revealing an important role of miR-210 in tumor initiation and progression [58]. Table 1 presents the identified targets of miR-210, showing the oncogenic role of miR-210.

**Table 1:** Targets of miR-210 functioning as oncogene

**Symbol**	**Description**	**Related function**	**Involved cell type**
MNT [22]	MAX network transcriptional repressor	Regulate cell proliferation	HCT116
			HeLa
			HFF-pBABE
			ME-180
			786-O-pBABE
Casp8ap2 [31]	Caspase 8 associated protein 2	Regulate apoptosis	MSC
PTBP3/ROD1 [61]	Polypyrimidine tract binding protein 3/Regulator of differentiation 1	Regulate apoptosis	HEK-293
			HUVEC
E2F3 [32]	E2F transcription factor 3	Regulate apoptosis and cell proliferation	HPASMC
BNIP3 [36]	BCL2/adenovirus E1B 19 kDa interacting protein 3	Induce apoptosis	NPC
			PC12
AIFM3 [27]	Apoptosis inducing factor, mitochondrion associated, 3	Induce apoptosis	SMMC-7721
			HepG2
			HuH7
EFNA3 [41,64]	Ephrin-A3	Regulate angiogenesis	HUVEC
VMP1 [42]	Vacuole membrane protein 1	Regulate migration and invasion	SMMC-7721
			HuH-7
RAD52 [66]	RAD52 homolog (S. cerevisiae)	Involve in DNA repair	HeLa
			MCF-7
PTPN1 [68]	protein tyrosine phosphatase, non-receptor type 1	Regulate immune response	IGR-Heu
			NA-8
HOXA1 [68]	Homeobox A1	Regulate immune response	IGR-Heu
			NA-8
TP53I11 [68]	tumor protein p53 inducible protein 11	Regulate immune response	IGR-Heu
			NA-8

*Abbreviations*: *MSC* mesenchymal stem cell, *HPASMC* human pulmonary artery smooth muscle cell, *NPCs* neural progenitor cell, *HUVEC* human umbilical vein endothelial cell.

#### miR-210 promotes cancer cell proliferation

Sustaining proliferative capacity is a key hallmark of cancer cells which acquire such capacity through a number of ways: 1) they may produce growth factor ligands themselves and stimulate normal cells in tumor-associated stroma to supply various growth factors, 2) they may harbor activating mutations to sustain proliferative signaling, and 3) they may disrupt negative-feedback loops that attenuate proliferative signaling [59].

Over the past few years, the role of hypoxia-inducible miR-210 in cell cycle regulation has been a subject of considerable interest and has provided reliable evidence supporting a role for miR-210 in promoting cancer cell proliferation [18,21,22,26-30]. Zhang and colleagues [22] identified MNT, a known MYC antagonist, as a miR-210 target. Overexpression of miR-210 can override hypoxia-induced cancer cell cycle arrest and promote cell proliferation by down-regulating MNT directly and activating c-MYC indirectly. Similarly but in a different way, Yang and colleagues [27] demonstrated that downregulation of miR-210 in hypoxic human hepatoma cells induced cell cycle arrest in the G0/G1, resulting in reduced cancer cell proliferation. However, functional targets of miR-210 contributing to such effect require further researches.

#### miR-210 inhibits apoptosis and protects cancer cell

Hypoxic cancer cells are notorious for their resistance to radiotherapy and many conventional chemotherapeutic agents, of which the underlying mechanisms remain to be revealed [3]. As the master HRM, the association of miR-210 and apoptosis as well as cell survival was intensively investigated. Its antiapoptotic and cytoprotective effects have been demonstrated in many studies involving not only cancer cells [27,60,61] but also normal cells such as human pulmonary artery smooth muscle cells (HPASMC) [32], cardiomyocytes [24,33], bone marrow-derived mesenchymal stem cells (MSCs) [31], as well as neural progenitor cells [36].

Many functional targets of miR-210 associated with apoptosis have been identified, as shown in Table 1. By downregulating the expression of caspase-8-associated protein-2 (Casp8ap2), miR-210 promoted the survival of MSCs that underwent ischemic preconditioning [31]. Through repressing the expression of regulator of differentiation 1 (ROD1), which is also named polypyrimidine tract binding protein 3 (PTBP3), miR-210 reduced the apoptosis of hypoxic cells and increased the survival of hypoxic cells [61]. E2F3, a member of the E2F family of transcriptional factors and a well-known cell cycle regulator, was identified as a direct target of miR-210 in hypoxic HPASMC, its downregulation was shown to be responsible in part for the antiapoptotic effect of miR-210 [32]. Knock down of miR-210 in hypoxic HPASMC, which resulted in concomitant upregulation of E2F3, induced apoptosis without significant change of cell proliferation, indicating the proapoptotic effect of E2F3 as well as the antiapoptotic effect of miR-210 in HPASMC under hypoxia stress [32]. The cytoprotective effect of miR-210 against radiotherapy was also investigated. Overexpression of miR-210 in A549 cell line (non-small cell lung carcinoma-derived cell line) under normoxia can protect cancer cells from radiation [57], while downregulation of miR-210 in hypoxic human hepatoma cells led to increased radiosensitivity, both in vitro and in vivo [27,62]. As elucidated by Grosso et al., A549 cells stably expressing miR-210 in normoxia exhibited similar radioresistance to A549 cells expressing miR-control in hypoxia, and hypoxia can further increase this resistance. Although the underlying mechanisms responsible for this radioresistance were not fully investigated, the authors gave us a clue that miR-210 expressing cells showed rapid repair of DNA double-strand breaks (DSBs), which might contribute to radioresistance [57]. In addition, synthetic miRNA-Mowers targeting miR-210 in bladder cancer cells can inhibit growth and migration and induce apoptosis [60].

#### miR-210 regulates angiogenesis, promotes invasion and metastasis

Inducing angiogenesis is another hallmark of cancer, which not only provides nutrients and oxygen, evacuates metabolic wastes and carbon dioxide to sustain cancer cells, but also facilitates metastasis [59]. Many miRNAs have been involved in tumor angiogenesis [44,63], including miR-21, miR-106a, miR-126, miR-155, miR-182, miR-210 and miR-424.

miR-210 overexpression in normoxic endothelial cells stimulated the formation of capillary-like structures and vascular endothelial growth factor-driven cell migration, while blockade had the opposite effect [41]. Ephrin-A3 (EFNA3) was identified as the direct target, whose down-modulation was necessary for miR-210 mediated stimulation of both tubulogenesis and chemotaxis [41]. Notably, hypoxia can increase the expression of EFNA3 mRNA, so the down-modulation of EFNA3 may attribute to translation inhibition [41]. Another study confirmed EFNA3 as a direct target of miR-210 through luciferase assay, however, upregulation of EFNA3 was shown in ischemia brain, which seemed to be contradictory with the hypothesis that hypoxia induced miR-210 expression would result in downregulation of EFNA3 [64]. Apparently, the unpredictable effects of miR-210 on the expression of EFNA3 need further investigation.

In hypoxic hepatocellular carcinoma (HCC), vacuole membrane protein 1 (VMP1) was identified as the direct and functional downstream target of miR-210, which mediates hypoxia-induced HCC cell migration and invasion [42]. Overexpression of miR-210 in non-invading breast cancer cell line MCF-7 cells led to cell invasion while repression of miR-210 in migrating and invading breast cell line MDA-MB-231 cells resulted in decreased cell migration and invasion [49]. Meanwhile, miR-210 contained in exosomes released by cancer cells can be transported to endothelial cells to induce angiogenesis [50].

#### miR-210 involves in DNA repair

Genome integrity is of vital importance for normal cells since mutations of crucial genes result in multiple diseases including cancer. Various stresses, including mutagens, ROS, ultraviolet light, radiation as well as chemotherapeutic agents can induce DNA damage, of which DNA double-strand break (DSB) has the most severe effect [65].

Cancer is characterized by genomic instability [59], which may result from hypoxic tumor microenvironment by affecting DNA repair capacity of cancer cells [5]. RAD52, a protein important for DNA DSB repair and homologous recombination, has been identified as a functional target of miR-210 [66]. In addition to down-regulating DNA repair genes, such as RAD52, to increase genomic instability which confers aggressiveness to cancer cells, miR-210 appears to be capable of facilitating DNA DSB repair after radiation exposure, which makes cancer cells more intractable, as demonstrated by Grosso et al. [57]. NSCLC cell lines expressing miR-210 in normoxia are more resistant to radiation due to more effective DNA repair, of which the underlying mechanism remains to be elucidated.

#### miR-210 induces immunosuppression

During the initiation and development of cancer, cancer cells have acquired multiple mechanisms to evade immunological surveillance. Emerging evidence has shown that certain miRNAs regulate expression of genes that are critically involved in both innate and adaptive immune responses [67]. A recent study investigated the role of miR-210, up-expressed in the hypoxic zones of human tumor tissues, in inducing immunosuppression in hypoxic cancer cells [68]. They examined the susceptibility of IGR-Heu (human NSCLC cell line) and NA-8 (human melanoma cell line) cells in which miR-210 expression had been abrogated by anti-miR-210 to cytotoxic T cells (CTC)-mediated lysis under hypoxia, demonstrated that these cancer cells were more susceptible to CTC-mediated lysis, implying the immunosuppressive effects of miR-210 in hypoxic cancer cells. Functional analysis has identified the potential targets of miR-210, including PTPN1, HOXA1 and TP53I11 that confer immunosuppression to hypoxic cells [68].

### miR-210 functions as a tumor suppressor

Controversial to the above cited multiple studies showing that miR-210 acts as an oncogene, many studies suggest that miR-210 can also act as a tumor suppressor, inhibiting tumor initiation. Table 2 summarizes the identified targets of miR-210, implying its potential role as tumor suppressor.

**Table 2:** Targets of miR-210 functioning as tumor suppressor gene

**Symbol**	**Description**	**Related function**	**Involved cell type**
E2F3 [18,21]	E2F transcription factor 3	Regulate apoptosis and cell proliferation	HeLa
			ACHN
			786-O
			Caki2
			HEK293
FGFRL1 [19,26]	fibroblast growth factor receptor-like 1	Regulate cell proliferation	MCF10A
			KYSE-170
			KYSE-590
PTPN2 [30]	protein tyrosine phosphatase, non-receptor type 2	Regulate cell proliferation	ASC
PIK1 [29]	1-phosphatidylinositol 4-kinase	Regulate mitosis	CNE
			HeLa
Cdc25B [29]	cell division cycle 25B	Regulate mitosis	CNE
			HeLa
Bub1B [29]	BUB1 mitotic checkpoint serine/threonine kinase B	Regulate mitosis	CNE
			HeLa
CCNF [29]	cyclin F	Regulate mitosis	CNE
			HeLa
Fam83D [29]	family with sequence similarity 83, member D	Regulate mitosis	CNE
			HeLa
Bcl-2 [34]	B-cell CLL/lymphoma 2	Apoptosis	Neuro-2a

*Abbreviations*: *ASC* adipose-derived stem cell.

#### miR-210 induces cell cycle arrest, inhibits cell proliferation, promotes apoptosis

In the study conducted by Giannakakis et al. [18], they found that miR-210 was deleted in 50% of ovarian cancer cell lines and 64% of ovarian cancer samples tested, implying miR-210 as a potential tumor suppressor gene. Subsequent functional analysis identified several targets of miR-210, including E2F3. Contradictory to our foregoing evidence of proapoptotic effect of E2F3 in hypoxia HPASMC, E2F3 was considered as a promoter of cell proliferaion here. Overexpression of miR-210 down-regulated E2F3 expression at the translational level, suggesting that down-regulation of miR-210 expression (such as demonstrated in ovarian cancer due to gene copy aberrations) in hypoxia may increase the expression of E2F3 that promotes cell proliferation and involves in tumorigenesis [18]. However, considering that E2F3 comprises two functionally different forms, E2F3a and E2F3b, with the same 3’ UTR, both E2F3a and E2F3b are targets of miR-210 [18], this interpretation warrants more experiments.

Tsuchiya et al. [26] also demonstrated the anti-proliferative role of miR-210 in cancer. They reported the down-expression of miR-210 in human esophageal squamous cell carcinoma (ESCC) and derived cell lines, and elucidated that overexpression of miR-210 in KYSE-170 (ESCC) cell line not only induces cell cycle arrest in both G0/G1 and G2/M phases, but also causes cell apoptosis and necrosis. Functional analysis identified fibroblast growth factor receptor-like 1 (FGFRL1) as the direct target.

Additional evidence has implicated miR-210 in mitotic regulation. In CNE cells treated with hypoxia mimetic agent, over-expression of exogenous miR-210 significantly decreased cell proliferation, and vice versa [29]. Molecular mechanism analysis revealed that a group of mitosis-related genes, including Plk1, Cdc25B, Cyclin F, Bulb1B and Fam83D, are the direct targets of miR-210, suggesting its inhibitory role on tumor formation.

In addition to inhibiting apoptosis as shown previously, miR-210 can mediate hypoxia-induced apoptosis at least in neuroblastoma cells as demonstrated by Chio et al. [34]. They treated neuro-2a (neuroblastoma cell line) cells with oxygen/glucose deprivation (OGD), elucidated the important role of miR-210 in OGD-induced cell apoptosis, and identified Bcl-2 as the functional target. Overexpression of miR-210 decreased the mRNA and protein levels of Bcl-2, an anti-apoptotic gene, resulting in increased apoptosis.

### miR-210 and mitochondrial metabolism

Under hypoxic conditions, cell metabolism shifts from mitochondrial oxidative phosphorylation to glycolysis (the Pasteur effect). HIF-1 plays a critical role in this effect, by up-regulating the expression of most glycolytic enzymes as well as pyruvate dehydrogenase kinase, while down-regulating mitochondrial respiration [69]. As tumors largely rely on glycolysis even under normal oxygen supply (Warburg effect) [59,70] which is significantly different from normal cells, the underling molecular mechanisms deserve further investigation.

The regulation of mitochondrial metabolism during hypoxia by miR-210 was first reported by Chan et al. [52]. They utilized pulmonary arterial endothelial cells as a representative hypoxic cell type, demonstrated that miR-210 directly targets iron-sulfur cluster assembly proteins (ISCU1/2) and decreases the activity of prototypical iron-sulfur proteins controlling mitochondrial metabolism, including ComplexIand acontiase, resulting in decreased oxidative phosphorylation. Subsequent studies investigating the role of miR-210 in modulating mitochondrial function have revealed more targets of miR-210 [53-57]. Besides ISCU [54], which was further confirmed, GPD1L [20], COX10 [53], SDHD and NDUFA4 [55] were also identified as direct targets involved in mitochondrial function regulation. In the study by Puissegur et al. [55], A549 cells overexpressing miR-210 exhibited an aberrant mitochondrial phenotype, mRNA expression profiling analysis linked miR-210 to mitochondrial dysfunction. Interestingly, miR-210 acts not only as a downstream mediator of HIF-1α, it can also promote HIF-1α stability by suppressing GPD1L, producing a positive feedback between HIF-1α and miR-210 [20]. As miR-210 is highly stable, when hypoxic cells undergo reoxygenation, HIF-1α is degraded immediately, but miR-210 remains stable to sustain glycolytic phenotype and inhibit mitochondrial metabolism under normoxia. Such advantage may be utilized by cancer cells, contributing to Warburg effect [57]. Taken together, the above evidence suggests an indisputable role of miR-210 in modulating mitochondrial metabolism, and facilitating adaptation of cancer cells to hypoxic condition.

### miR-210 as diagnostic and prognostic biomarker in cancer

Early diagnosis and prognosis evaluation of cancer are of vital importance to improve treatment outcome. It is well acknowledged that cancer cells or tissues harbor aberrant miRNA expression profiles compared to normal cells or tissues, and specific miRNA signature can not only be used for diagnosis but also to classify cancer patients into subgroups with different prognosis guiding individualized treatment [71-77]. Many studies have investigated the role of miR-210 in cancer diagnosis and prognosis, however, presenting apparently conflicting results.

Most evidence showed that miR-210 was up-regulated in many solid tumors, including breast cancer [16,78-80], head and neck cancer [17,76], pancreatic cancer [81-83], lung cancer [55,84-87], renal cancer [23,88,89], lymphoma [90], osteosarcoma [91], esophageal cancer [92] as well as ovarian cancer [93]. There are also some inconsistent evidence that miR-210 was deleted in some cases of ovarian cancer [18], and was down-regulated in some cases of esophageal cancer [26], exhibiting the complexity and heterogeneity of cancer.

Table 3 enumerates the studies [81,86,94-100] investigating the diagnostic value of miR-210, either alone or in combination with other miRNAs, providing the sensitivity and specificity of miR-210 when it was used alone to discriminate cancer from non-cancer. Because of the inherent stability of miRNAs [101], and the high sensitivity as well as specificity of quantitative RT-PCR [102], samples used for diagnosis include not only tumor tissue but also serum, plasma, sputum and stool that are more available in clinic, showing the promise of miRNAs in clinical application [103]. Notably, the diagnostic power increased when using multiple miRNAs instead of only one miRNA [81,86,94-97]. For example, in the study conducted by wang et al. [81], they profiled four pancreatic cancer related miRNAs (miR-21, miR-210, miR-155, miR-196a) as blood-based biomarker for diagnosis. The sensitivity and specificity were 42% to 53% and 73% to 89% respectively, when using only one miRNA for diagnosis, but with the panel of four miRNAs, the sensitivity and specificity increased to 64% and 89% respectively. Other similar studies also showed us similar results [86,94-97].

**Table 3:** Studies investigating diagnostic value of miR-210

**First author**	**Publication year**	**Types of cancer**	**Types of sample**	**Negative controls**	**Sensitivity**	**Specificity**
Wang [81]	2009	Pancreatic cancer	plasma	Healthy controls	53%	78%
Xing [86]	2010	Squamous cell LC	sputum	Healthy controls	58%	79%
Shen [94]	2011	Lung cancer	plasma	Benign SPNs	56%	73%
Tan [95]	2011	Squamous cell LC	tissue	Normal lung tissue	Not provided	Not provided
Ren [96]	2012	Pancreatic cancer	stool	Healthy controls	85%	67%
Li [97]	2013	NSCLC	sputum	Healthy controls	Not provided	Not provided
Li [98]	2013	NSCLC	serum	Healthy controls	79%	74%
Zhao [99]	2013	Renal cancer	serum	Healthy controls	81%	79%
Iwamoto [100]	2014	Renal cancer	serum	Healthy controls	65%	83%

*Abbreviations*: *LC* lung cancer, *NSCLC* non-small cell lung cancer, *SPN* solitary pulmonary nodule.

Table 4 lists the studies [16,17,23,78-80,82,87,90,91,104-107] investigating the prognostic value of miR-210. While most studies documented that high miR-210 expression level in tumor tissue or blood was correlated with poor disease-free and/or overall survival and was a negative prognostic factor, at least three articles investigating soft-tissue sarcoma [104], renal cancer [23] and NSCLC [87] respectively, indicated that miR-210 was a positive prognostic factor. Obviously, the prognostic value of miR-210 expression level in specific cancer type with specific stage varies, and needs more exploration. The interesting study by Buffa et al. presented us an excellent example for exploring miRNAs as prognostic factors for cancer. They conducted comprehensive miRNA and mRNA expression profiling in a large cohort of 207 early-invasive breast cancers. To identify miRNAs with independent prognostic value, they performed penalized Cox regression for distant relapse-free survival (DRFS), including all miRNAs, clinical covariates and gene signatures. At last, they detected four microRNAs to be independently associated with DRFS in estrogen receptor (ER)-positive and six in ER-negative (including miR-210) cases. To further confirm the value of these prognostic miRNAs, they also carried out correlation analysis of these miRNAs with clinicopathologic factors, indicating that miR-210 expression was associated with proliferation, ER positivity, grade, and nodal invasion. In addition, they performed prognostic analysis of pri-miRNAs and predicted target transcripts of prognostic miRNAs, as well as miRNA-processing genes, revealing that identified miRNAs were virtually independent prognostic factors. They also demonstrated that combination of miRNA and target expression could identify patients with the poorest prognosis, showing us the prospect of integrating miRNA and mRNA information for prognosis analysis.

**Table 4:** Studies investigating prognostic value of miR-210

**First author**	**Publication year**	**Types of cancer**	**Types of sample**	**RR or HR (high VS low expression level)**
Camps [16]	2008	Breast cancer	tissue	4.07(PFS), 11.38(OS)
Lawrie [90]	2008	Diffuse large B-cell lymphoma	serum	No significance
Gee [17]	2010	Head and neck cancer	tissue	Not provided
Greither [82]	2010	Pancreatic cancer	tissue	2.48
Buffa [107]	2011	Breast cancer (ER^−^)	tissue	Not provided
Radiojicic [78]	2011	Triple-negative breast cancer	tissue	No significance
Rothe [80]	2011	Breast cancer	tissue	4.43(RFS)
Greither [104]	2012	Soft-tissue sarcoma	tissue	3.19(PFS)^*^
Toyama [79]	2012	Breast cancer	tissue	4.39
Volinia [105]	2012	Breast cancer	tissue	1.54(OS)
Cai [91]	2013	Pediatric osteosarcoma	tissue	2.6(PFS), 3.3(OS)
Eilertsen [87]	2013	Non-small cell lung cancer	tissue^#^	1.9(DSS)^**^
McCormick [23]	2013	Renal cancer	tissue	Not provided
Qiu [106]	2013	Glioblastoma	tissue	0.75^**^

^*^intermediate VS high expression level.

^#^stromal cells in tumor tissues.

^**^low VS high expression level.

*Abbreviations*: *PFS* progression-free survival, *OS* overall survival, *RFS* relapse-free survival, *DSS* disease-specific survival, *ER*^*−*^ estrogen receptor negative.

### Conclusions and future directions

As the master HRM, regulated mainly by HIF-1, miR-210 plays an essential role in hypoxic response. In addition to regulating mitochondrial metabolism, miR-210 is involved in regulating cell cycle, cell survival, differentiation, DNA repair as well as immune response.

Since hypoxia can influence both cell death and survival [108], it is not surprising that miR-210 can act both as an oncogene and a tumor suppressor, depending on cellular context, the extent and duration of hypoxia. A reasonable explanation is that since miRNAs can target hundreds of mRNAs with differential biological functions, the ultimate effect of miR-210 depends on the target mRNAs that are available in certain cells. In addition to multiple targets discussed in this review, many other genes have been identified as miR-210 targets, and more and more potential target genes are emerging [12]. An alternative possibility may be that miR-210 acts as a tumor suppressor at the beginning of tumorigenesis when hypoxia is not significant. However, with the progression of tumor, hypoxia becomes significant, tumor cells evolve, become resistant to hypoxia and adapt well to highly expressed miR-210, then miR-210 switches to an oncogene [19,29]. Due to the paradoxical functions of miR-210, more investigations are warranted before considering it as an effective therapeutic target in cancer. Future researches should elucidate the specific context that is responsible for specific functions of miR-210. In addition, how to integrate multiple functionally different but related targets of one peculiar miRNA such as miR-210, so as to precisely predict its functions remains a great challenge.

Besides functions of miR-210, we also reviewed the diagnostic and prognostic value of it. As described above, up-regulated miR-210 is not only be detected in cancer tissues, but also in body fluids. It is feasible to discriminate cancer from non-cancer with a specific group of miRNAs including miR-210. However, when it comes to prognosis, it is far too early to use miR-210 alone as a prognostic factor without dispute, and more investigations are needed to elucidate the underlying mechanism of such discrepancy. In future, global analysis of large cohorts of patients with not only miRNAs expression profile but also mRNAs expression profile, even integrated with other genetic information such as DNA copy number variance, single nucleotide polymorphisms, will provide us more insights about significant prognostic factors as well as novel therapeutic targets.

## Competing interests

The authors declare that they have no competing interests.

## Authors’ contributions

QQ and LBS conceived the study. QQ and WFR searched the literature and drafted the manuscript. LBS edited the manuscript. All the authors have read and approved the final manuscript.
